# Supplementary material for: Association between Angiotensin-converting enzyme (ACE) insertion/deletion polymorphism and hypertension in a Ghanaian population
Source: PLoS One. 2024 Dec 12;19(12):e0311692. doi: 10.1371/journal.pone.0311692 (PMC11637408; doi:10.1371/journal.pone.0311692)
Supplement: S1 Raw images — (PDF) [file pone.0311692.s003.pdf]

Primer sequence and expected amplicon sizes.

Polymorphism	Primer sequence	Genotype	Amplicon size(bp)
I/D			
Forward	5’-CTGGAAGAGACCACTCCCATCCTTTCT’-3	DD	190
Reverse	5’GATGTGGCCATCACATTCGTCAGAT3’	II	490
		ID	490 + 190

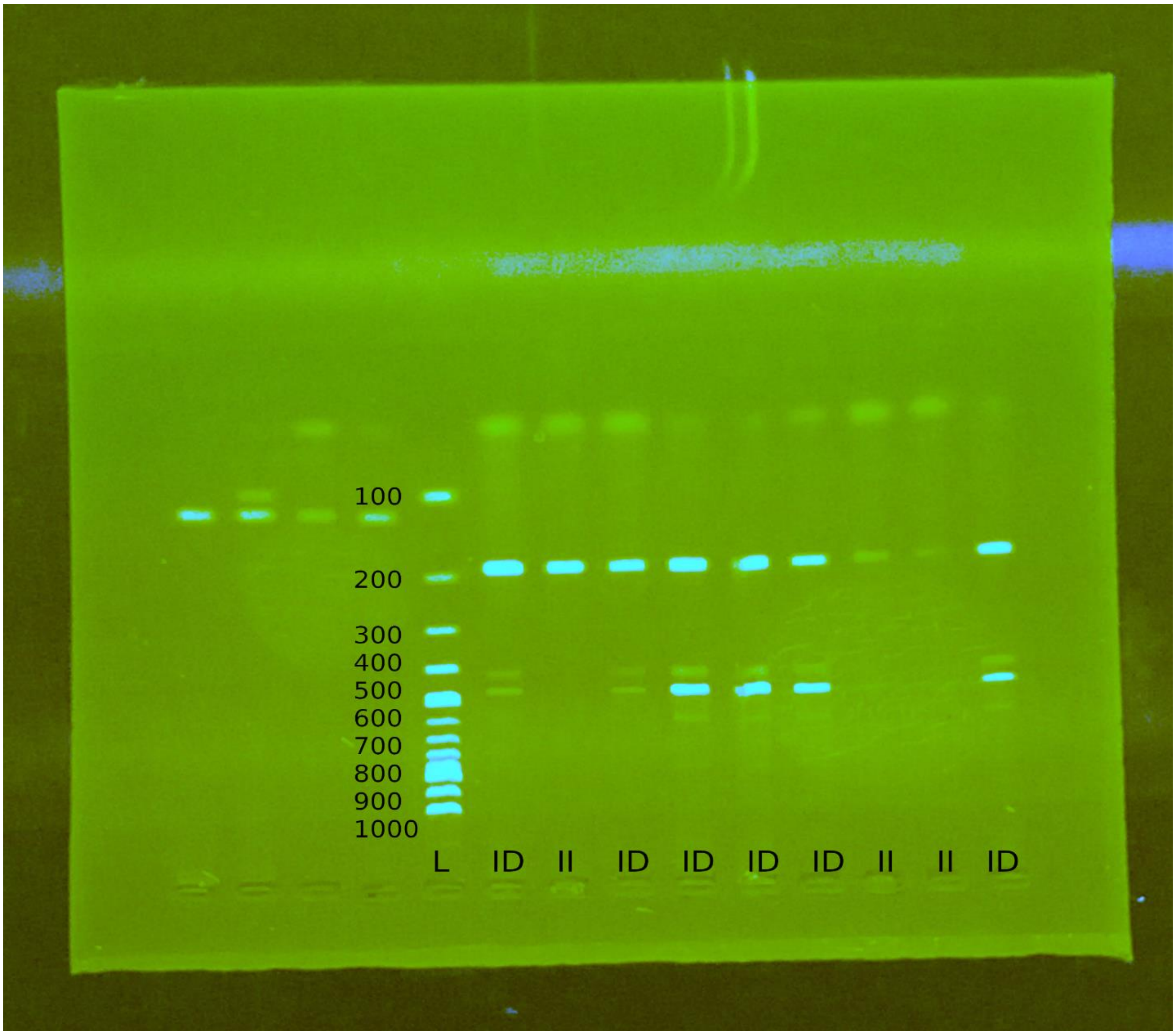

Gel Images for Hypertensive Patients

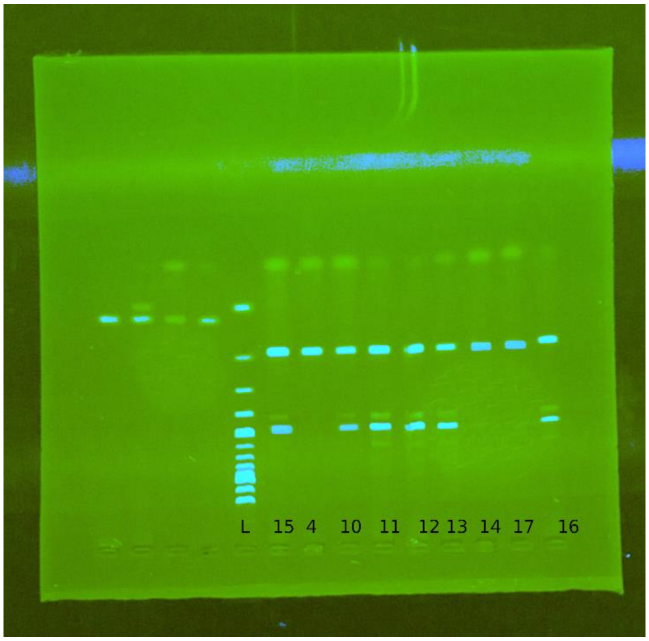

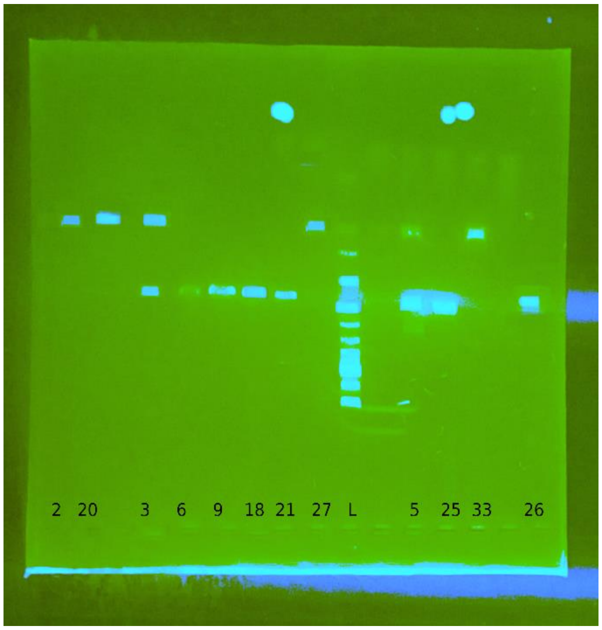

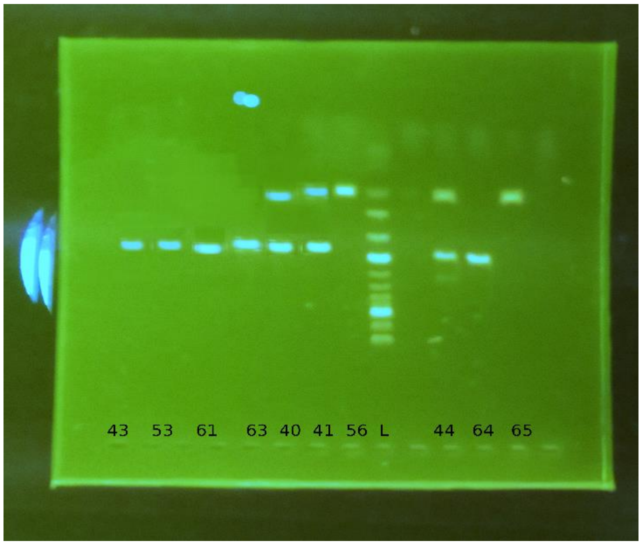

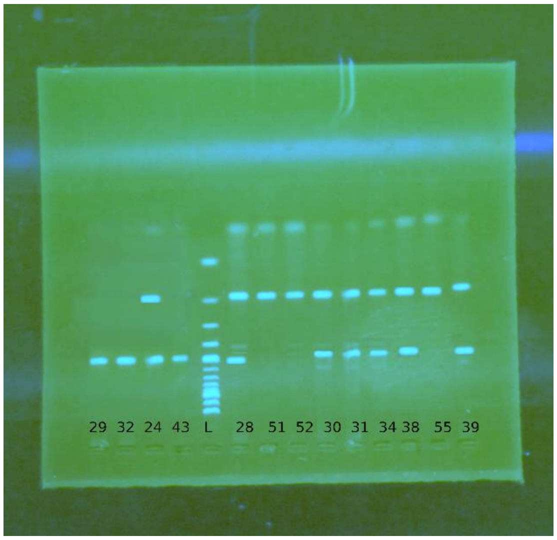

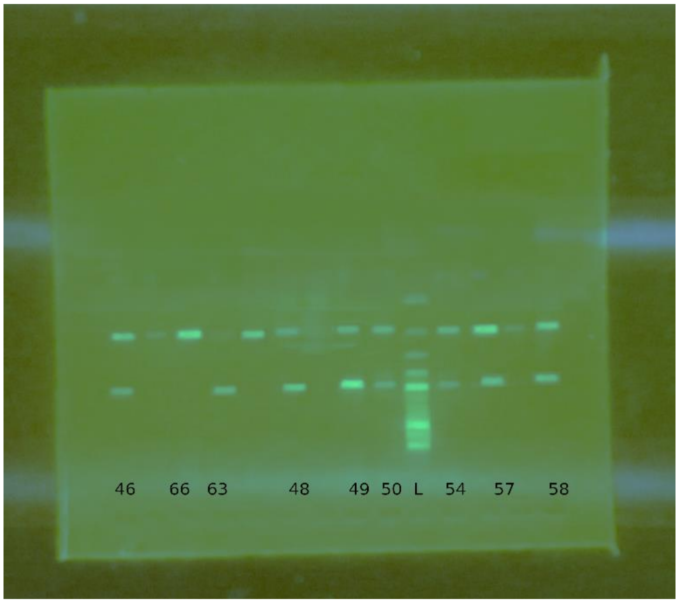

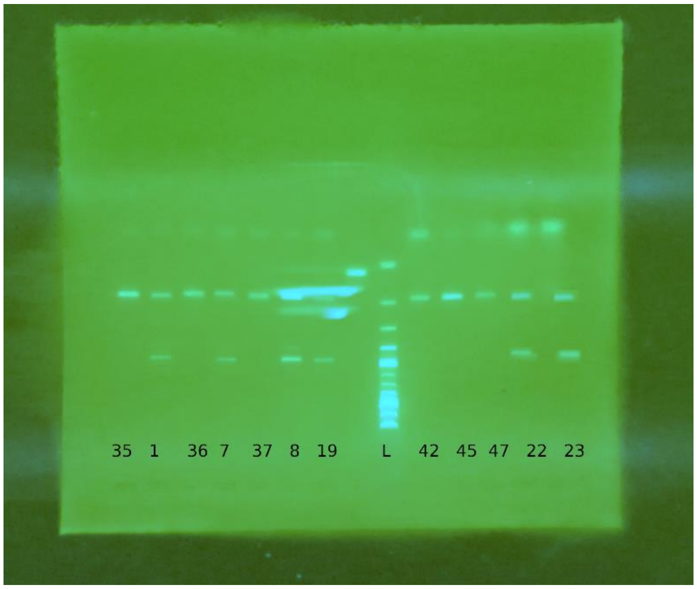

Gel images for Normotensive Individuals

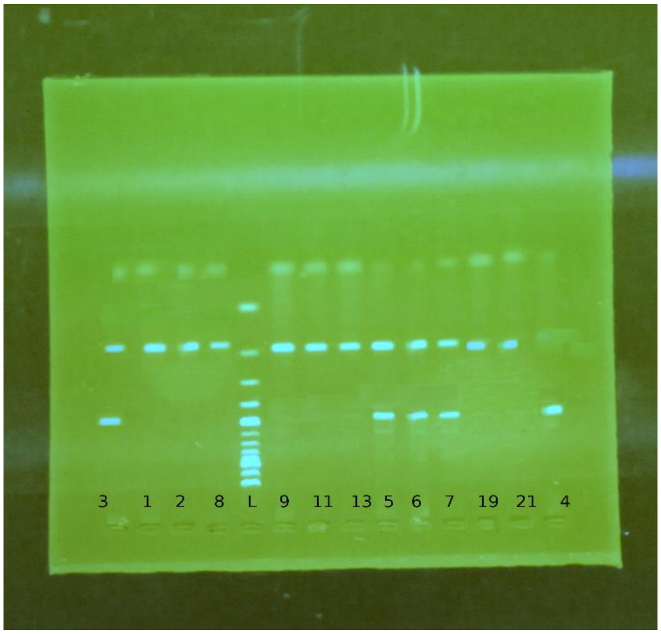

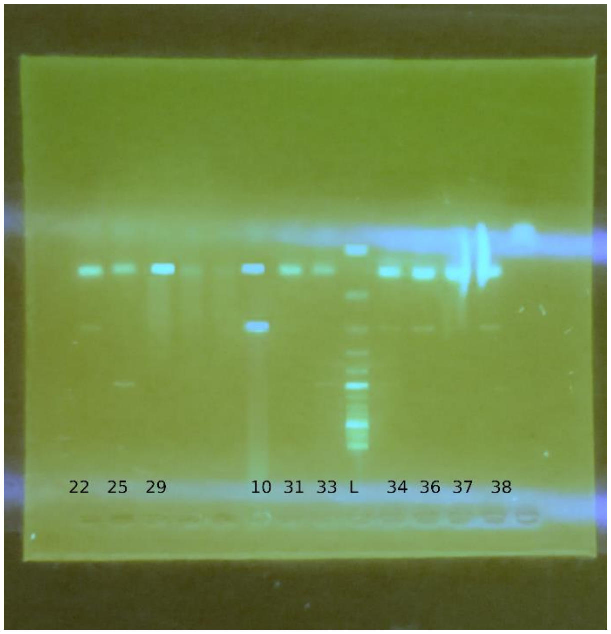

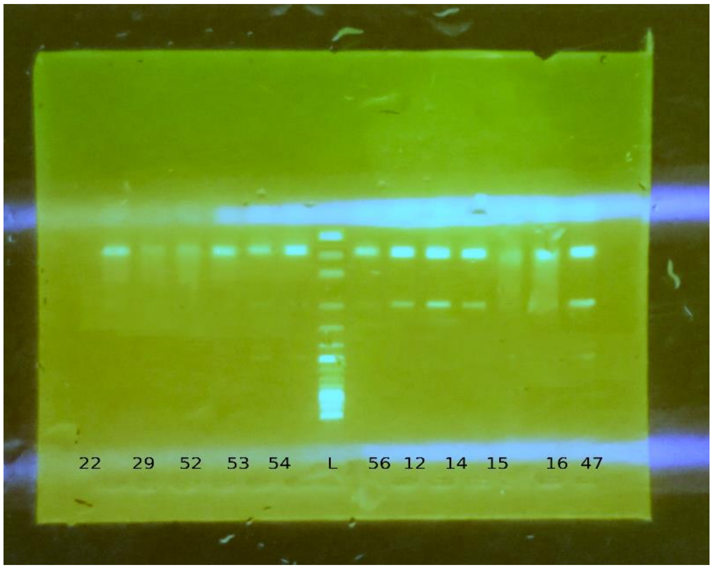
22 29 52 53 54 L 56 12 14 15 16 47

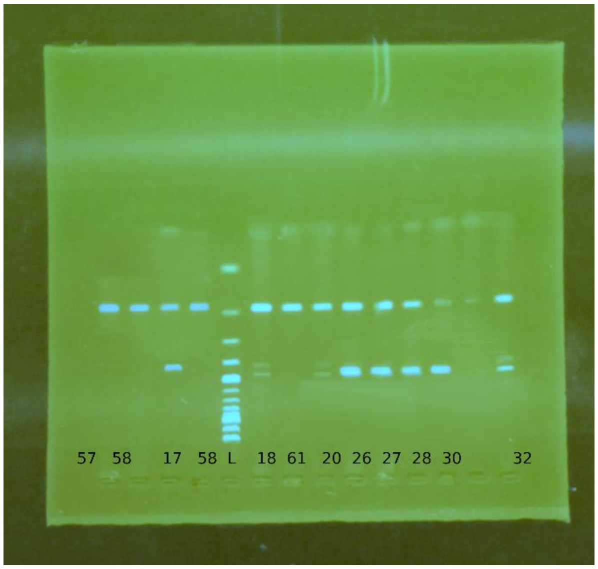
57 58 17 58 L 18 61 20 26 27 28 30 32

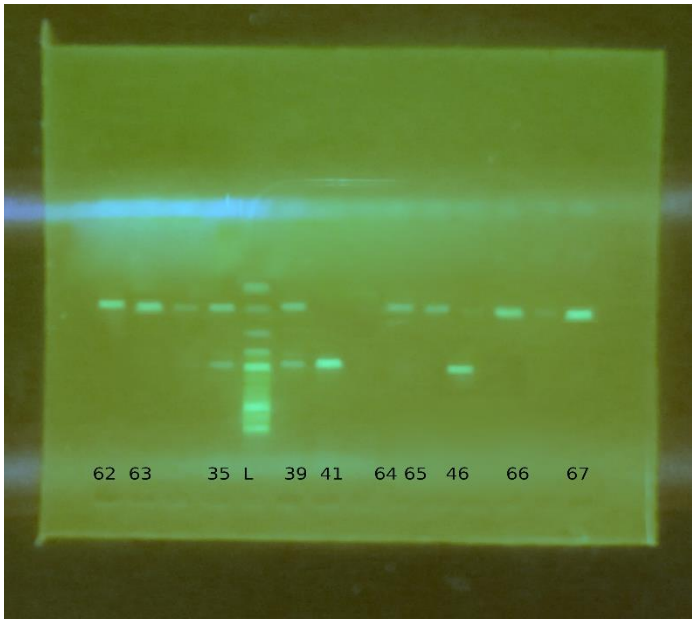
62 63 35 L 39 41 64 65 46 66 67

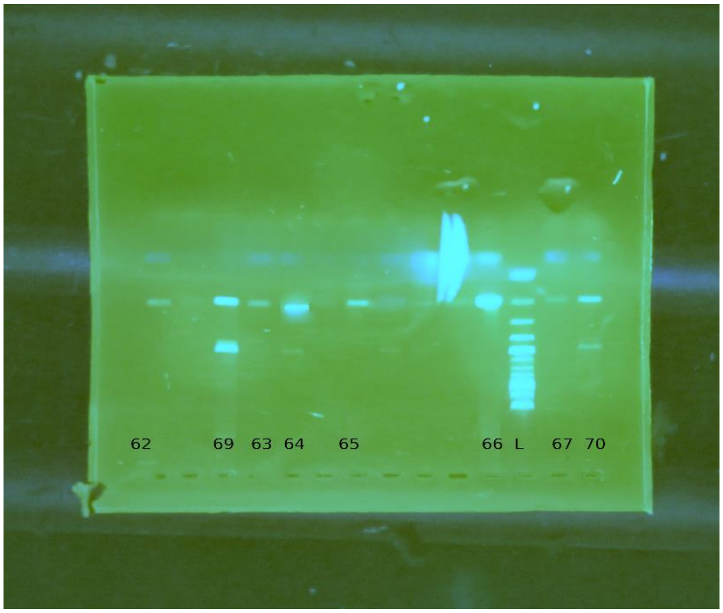
62 69 63 64 65 66 L 67 70
