# Supplementary material for: Association between Angiotensin-converting enzyme (ACE) insertion/deletion polymorphism and hypertension in a Ghanaian population
Source: PLoS One. 2024 Dec 12;19(12):e0311692. doi: 10.1371/journal.pone.0311692 (PMC11637408; doi:10.1371/journal.pone.0311692)
Supplement: S1 File — (DOC) [file pone.0311692.s004.doc]

**WHO STEPS Instrument**

**Question-by-Question Guide**

**(Core and Expanded)**


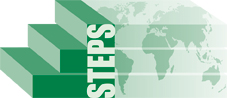


The WHO STEPwise approach to noncommunicable disease risk factor surveillance (STEPS)


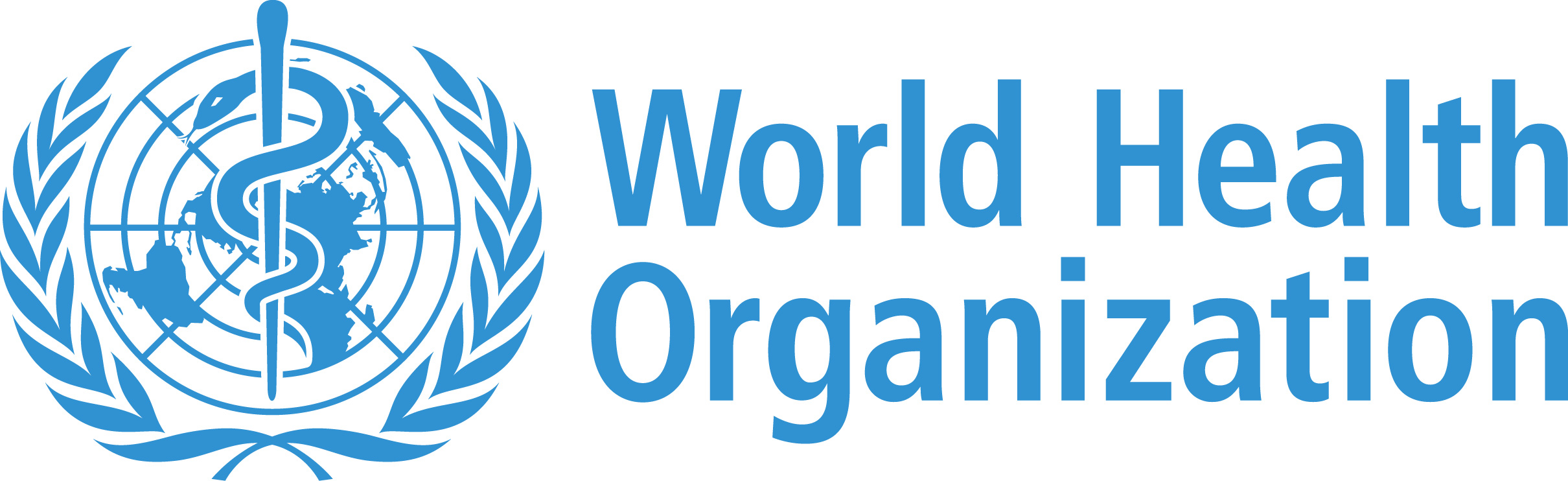
World Health Organization

20 Avenue Appia, 1211 Geneva 27, Switzerland

*For further information:* [www.who.int/ncds/steps](http://www.who.int/ncds/steps)

# STEPS Question-by-Question (Q-by-Q) Guide

#### Overview

| Introduction | The Question-by-Question Guide presents the STEPS Instrument with a brief explanation for each of the questions. |
| --- | --- |

| Purpose | The purpose of the Question-by-Question Guide is to provide background information to the interviewers and supervisors as to what is intended by each question.  Interviewers can use this information when participants request clarification about specific questions or they do not know the answer. |
| --- | --- |
|  | Interviewers and supervisors should refrain from offering their own interpretations. |

| Guide to the columns | The table below is a brief guide to each of the columns in the Q-by-Q Guide. |
| --- | --- |

| Column | Description | Site Tailoring |
| --- | --- | --- |
| Question | Each question is to be read to the participants | - Select sections to use. - Add expanded and optional questions as desired. |
| Response | This column lists the available response options which the interviewer will be circling or filling in the text boxes. The skip instructions are shown on the right hand side of the responses and should be carefully followed during interviews. | - Add site specific responses for demographic responses (e.g. C6). - Change skip question identifiers where necessary. |
| Code | The column is designed to match data from the instrument into the data entry tool, data analysis syntax, data book, and fact sheet. | This should never be changed or removed. The code is used as a general identifier for the data entry and analysis. |


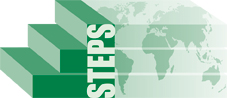
WHO STEPS Q-by-Q Guide

for Noncommunicable Disease
Risk Factor Surveillance

<insert country/site name>

| **Survey Information** |
| --- |

| Location and Date | | Response | | Code | |
| --- | --- | --- | --- | --- | --- |
| Cluster/Centre/Village ID*Enter Cluster, Centre or Village ID from list provided.* | └─┴─┴─┴─┴─┴─┘ | | I1 | |  |
| Cluster/Centre/Village name*Enter Cluster, Centre or Village name as appropriate.* |  | | I2 | |  |
| Interviewer ID*Enter interviewer's identification.* | └─┴─┴─┘ | | I3 | |  |
| Date of completion of the instrument*Enter date when instrument actually completed.* | └─┴─┘ └─┴─┘ └─┴─┴─┴─┘ dd mm year | | I4 | |  |

| Consent, Interview Language and Name | Response | | Code |
| --- | --- | --- | --- |
| Consent has been read and obtained  *Select relevant response.* | Yes | 1 | I5 |
|  | No | 2 **If NO, END** |  |
| Interview Language *[Insert Language]*  *Select relevant response.* | English | 1 | I6 |
|  | *[Add others]* | 2 |  |
|  | *[Add others]* | 3 |  |
|  | *[Add others]* | 4 |  |
| Time of interview  (24 hour clock)  *Enter time interview started.* | └─┴─┘: └─┴─┘  hrs mins | | I7 |
| Family Surname  *Enter family surname (reassure the participant on the confidential nature of this information and that this is only needed for follow up).* |  | | I8 |
| First Name  *Enter first name of respondent (reassure the participant on the confidential nature of this information and that this is only needed for follow up).* |  | | I9 |
| **Additional Information that may be helpful** | | | |
| Contact phone number where possible  *Enter phone number (reassure the participant on the confidential nature of this information and that this is only needed for follow up).* |  | | I10 |

| **Step 1 Demographic Information** |
| --- |

| **CORE: Demographic Information** | | | | |
| --- | --- | --- | --- | --- |
| **Question** | **Response** | | | **Code** |
| Sex (*Record Male / Female as observed)*  *Select Male / Female as observed.* | Male | | 1 | C1 |
|  | Female | | 2 |  |
| What is your date of birth?  *Don't Know 77 77 7777*  *Enter date of birth of participant. If unknown, select “don’t know”.* | └─┴─┘ └─┴─┘ └─┴─┴─┴─┘ *If known, Go to C4*  dd mm year | | | C2 |
| How old are you?  *If the age is unknown, help participant estimate their age by interviewing them about their recollection of widely known major events.* | Years | | └─┴─┘ | C3 |
| In total, how many years have you spent at school and in full-time study (excluding pre-school)?  *Enter total number of years of education (excluding pre-school and kindergarten).* | Years | └─┴─┘ | | C4 |

| **EXPANDED: Demographic Information** | | | | |
| --- | --- | --- | --- | --- |
| What is the **highest level of education** you have completed?  *[INSERT COUNTRY-SPECIFIC CATEGORIES]*  *If a person attended a few months of the first year of secondary school but did not complete the year, select “primary school completed”. If a person only attended a few years of primary school, select “less than primary school”.*  *Select appropriate response.* | No formal schooling | | 1 | C5 |
|  | Less than primary school | | 2 |  |
|  | Primary school completed | | 3 |  |
|  | Secondary school completed | | 4 |  |
|  | High school completed | | 5 |  |
|  | College/University completed | | 6 |  |
|  | Post graduate degree | | 7 |  |
|  | Refused | | 88 |  |
| What is your *[insert relevant ethnic group / racial group / cultural subgroup / others]* **background**?  *Select the relevant ethnic/cultural group to which the participant belongs.* | [*Locally defined]* | | 1 | C6 |
|  | [*Locally defined]* | | 2 |  |
|  | [*Locally defined]* | | 3 |  |
|  | Refused | | 88 |  |
| What is your **marital status**?  *Select the appropriate response.* | Never married | | 1 | C7 |
|  | Currently married | | 2 |  |
|  | Separated | | 3 |  |
|  | Divorced | | 4 |  |
|  | Widowed | | 5 |  |
|  | Cohabitating | | 6 |  |
|  | Refused | | 88 |  |
| Which of the following best describes your **main** **work** status over the past 12 months?  *[INSERT COUNTRY-SPECIFIC CATEGORIES]*  (*USE SHOWCARD)*  *The purpose of this question is to help answer other questions such as whether people in different kinds of occupations may be confronted with different risk factors.*  *Select appropriate response.* | Government employee | | 1 | C8 |
|  | Non-government employee | | 2 |  |
|  | Self-employed | | 3 |  |
|  | Non-paid | | 4 |  |
|  | Student | | 5 |  |
|  | Homemaker | | 6 |  |
|  | Retired | | 7 |  |
|  | Unemployed (able to work) | | 8 |  |
|  | Unemployed (unable to work) | | 9 |  |
|  | Refused | | 88 |  |
| How many people older than 18 years, including yourself, live in your household?  *Enter the total number of people living in the household who are 18 years or older.* | Number of people | | └─┴─┘ *If Not Known, Go to C11* | C9 |
| **EXPANDED: Demographic Information,** Continued | | | | |
| **Question** | **Response** | | | **Code** |
| Taking **the past year**, can you tell me what the average earnings of the household have been?  *(RECORD ONLY ONE, NOT ALL 3)*  *Enter the average earnings of the household by week, month, or year. If refused to answer, skip to C11.* | Per week | └─┴─┴─┴─┴─┴─┴─┘ *Go to T1* | | C10a |
|  | OR per month | └─┴─┴─┴─┴─┴─┴─┘  *Go to T1* | | C10b |
|  | OR per year | └─┴─┴─┴─┴─┴─┴─┘ *Go to T1* | | C10c |
|  | Refused | 88 | | C10d |
| Can you give an **estimate** of the annual household income if I read some options to you? Is it  *[INSERT QUINTILE VALUES IN LOCAL CURRENCY]*  *(READ OPTIONS)*  *Select the appropriate quintile value for the annual household income.* | ≤ Quintile (Q) 1 | | 1 | C11 |
|  | More than Q 1, ≤ Q 2 | | 2 |  |
|  | More than Q 2, ≤ Q 3 | | 3 |  |
|  | More than Q 3, ≤ Q 4 | | 4 |  |
|  | More than Q 4 | | 5 |  |
|  | Don't Know | | 77 |  |
|  | Refused | | 88 |  |

| **Step 1 Behavioural Measurements** |
| --- |

| **CORE: Tobacco Use** | | | |
| --- | --- | --- | --- |
| Now I am going to ask you some questions about tobacco use. | | | |
| **Question** | **Response** | | **Code** |
| Do you **currently** smoke any **tobacco** products, such as cigarettes, cigars or pipes?  *(USE SHOWCARD)*  *Ask the participant to think of any tobacco products he/she is smoking currently.* | Yes | 1 | T1 |
|  | No | 2 *If No, go to T8* |  |
| Do you currently smoke tobacco products **daily**?  *This question is only for current smokers of tobacco products.* | Yes | 1 | T2 |
|  | No | 2 |  |
| How old were you when you **first started** smoking?  *For current smokers only. Ask the participant to think of the time when he/she started to smoke any tobacco products.* | Age (years) | **└─┴─┘** *If Known, go to T5a/T5aw* | T3 |
|  | Don’t know 77 |  |  |
| Do you remember how long ago it was?  *(RECORD ONLY 1, NOT ALL 3)*  *Don’t know 77*  *If the participant doesn’t remember his/her age when started smoking, then record the time in years, months or weeks as appropriate.* | In Years | **└─┴─┘** *If Known, go to T5a/T5aw* | T4a |
|  | OR in Months | **└─┴─┘** *If Known, go to T5a/T5aw* | T4b |
|  | OR in Weeks | **└─┴─┘** | T4c |
| On average, **how many** of the following products do you smoke **each day/week?**  *(IF LESS THAN DAILY, RECORD WEEKLY)*  *(RECORD FOR EACH TYPE, USE SHOWCARD)*  *Don’t Know 7777*  *For current smokers only. Specify zero if no products were used in each category instead of leaving categories blank.*  *Record daily consumption for daily smokers. If products are smoked less than daily by daily smokers, enter weekly consumption. Also enter weekly consumption for current, non-daily smokers.* | DAILY**↓** WEEKLY↓ | | |
|  | Manufactured cigarettes | └─┴─┴─┴**─┘**└─┴─┴─┴**─┘** | T5a/T5aw |
|  | Hand-rolled cigarettes | └─┴─┴─┴**─┘**└─┴─┴─┴**─┘** | T5b/T5bw |
|  | Pipes full of tobacco | └─┴─┴─┴**─┘**└─┴─┴─┴**─┘** | T5c/T5cw |
|  | Cigars, cheroots, cigarillos | └─┴─┴─┴**─┘**└─┴─┴─┴**─┘** | T5d/T5dw |
|  | Number of Shisha sessions | └─┴─┴─┴**─┘**└─┴─┴─┴**─┘** | T5e/T5ew |
|  | Other | └─┴─┴─┴**─┘**└─┴─┴─┴**─┘** *If Other, go to T5other, else go to T6* | T5f/T5fw |
|  | Other (please specify): | └─┴─┴─┴─┴─┴─┘ | T5other/  T5otherw |
| During the past 12 months, have you tried to **stop smoking**?  *For current smokers only. Ask the participant to think of any quit attempt during the past 12 months.* | Yes | 1 | T6 |
|  | No | 2 |  |
| During any visit to a doctor or other health worker in the past 12 months, were you advised to quit smoking tobacco?  *For current smokers only. Ask the participant to think of visits to a doctor or other health worker during the past 12 months. If no visit, select “no visit during the past 12 months”.* | Yes | 1 *If T2=Yes, go to T12; if T2=No, go* *to T9* | T7 |
|  | No | 2 *If T2=Yes, go to T12; if T2=No, go to T9* |  |
|  | No visit during the past 12 months | 3 *If T2=Yes, go to T12; if T2=No, go to T9* |  |
| In the past, did you **ever** **smoke** any tobacco products?  *(USE SHOWCARD)*  *Ask the participant to think of the time when he/she may have been smoking tobacco products.* | Yes | 1 | T8 |
|  | No | 2 *If No, go to T12* |  |
| In the past, did you **ever** smoke **daily**?  *Ask the participant to think of the time when he/she may have been smoking tobacco products on a daily basis.* | Yes | 1 *If T1=Yes, go to T12, else go to T10* | T9 |
|  | No | 2 *If T1=Yes, go to T12, else go to T10* |  |

| **EXPANDED: Tobacco Use** | | | |
| --- | --- | --- | --- |
| **Question** | **Response** | | **Code** |
| How old were you when you **stopped** smoking?  *Ask the participant to think of the time when he/she stopped smoking tobacco products.* | Age (years) | **└─┴─┘** *If Known, go to T12* | T10 |
|  | Don’t Know 77 |  |  |
| How **long ago** did you stop smoking?  *(RECORD ONLY 1, NOT ALL 3)*  *Don’t Know 77*  *If the participant doesn't remember his/her age when they stopped smoking, then record the time in weeks, months or years as appropriate.* | Years ago | **└─┴─┘** *If Known, go to T12* | T11a |
|  | OR Months ago | **└─┴─┘** *If Known, go to T12* | T11b |
|  | OR Weeks ago | **└─┴─┘** | T11c |
| Do you **currently use** any **smokeless tobacco** products such as *[snuff, chewing tobacco, betel]*?  *(USE SHOWCARD)*  *Ask the participant to think of any smokeless tobacco products that he/she is using currently.* | Yes | 1 | T12 |
|  | No | 2 *If No, go to T15* |  |
| Do you **currently use** **smokeless tobacco** products **daily?**  *For current users of smokeless tobacco products only.* | Yes | 1 | T13 |
|  | No | 2 *If No, go to T14aw* |  |
| On average, how many **times a day/week** do you use ….  *(IF LESS THAN DAILY, RECORD WEEKLY)*  *(RECORD FOR EACH TYPE, USE SHOWCARD)*  *Don’t Know 7777*  *For current users of smokeless tobacco only.*  *Record for each type of smokeless tobacco products. Specify zero if no products were used in each category instead of leaving categories blank.*  *Record daily consumption for daily users. If products are used less than daily by daily users, enter weekly consumption. Also enter weekly consumption for current, non-daily users.* | DAILY**↓** WEEKLY↓ | | |
|  | Snuff, by mouth | └─┴─┴─┴**─┘**└─┴─┴─┴**─┘** | T14a/  T14aw |
|  | Snuff, by nose | └─┴─┴─┴**─┘**└─┴─┴─┴**─┘** | T14b/  T14bw |
|  | Chewing tobacco | └─┴─┴─┴**─┘**└─┴─┴─┴**─┘** | T14c/  T14cw |
|  | Betel, quid | └─┴─┴─┴**─┘**└─┴─┴─┴**─┘** | T14d/  T14dw |
|  | Other | └─┴─┴─┴**─┘**└─┴─┴─┴**─┘** *If Other, go to T14other, if T13=No, go to T16, else go to T17* | T14e/  T14ew |
|  | Other (please specify): | └─┴─┴─┴─┴─┴─┘  *If T13=No, go to T16, else go to T17* | T14other/  T14otherw |
| In the **past**, did you **ever use** smokeless tobacco products such as *[snuff, chewing tobacco, or betel]*?  *Ask the participant to think of the time when he/she may have been using smokeless tobacco products.* | Yes | 1 | T15 |
|  | No | 2 *If No, go to T17* |  |
| In the **past**, did you **ever use** smokeless tobacco products such as *[snuff, chewing tobacco, or betel]* **daily**?  *Ask the participant to think of the time when he/she may have been using smokeless tobacco products on a daily basis.* | Yes | 1 | T16 |
|  | No | 2 |  |
| During the past 30 days, did someone smoke **in your home**?  *The participant should only think about other people, not about him-/herself. Smokers should exclude themselves.*  *The question is asking about inside the participant’s home. This only includes fully enclosed areas of the home.* | Yes | 1 | T17 |
|  | No | 2 |  |
| During the past 30 days, did someone smoke in closed areas **in your workplace** (in the building, in a work area or a specific office)?  *For those not working in a closed area, record “don’t work in a closed area”.*  *Ask the participant to think of seeing somebody smoke or smelling the smoke in indoor areas at work during the past 30 days.* | Yes | 1 | T18 |
|  | No | 2 |  |
|  | Don't work in a closed area | 3 |  |

| **CORE: Alcohol Consumption** | | | |
| --- | --- | --- | --- |
| The next questions ask about the consumption of alcohol. | | | |
| **Question** | **Response** | | **Code** |
| Have you **ever** consumed any alcohol such as beer, wine, spirits or *[add other local examples]*?  *(USE SHOWCARD OR SHOW EXAMPLES)*  *Ask the participant to think of any alcohol, with the exception of alcohol-based medication that is taken due to health reasons. Even if the participant has only consumed a few sips of alcohol, the response should be “Yes”.* | Yes | 1 | A1 |
|  | No | 2  *If No, go to A16* |  |
| Have you consumed any alcohol within the **past 12 months**?  *Ask the participant to think of any alcohol, with the exception of alcohol-based medication that is taken due to health reasons. Even if the participant has only consumed a few sips of alcohol in the past 12 months, the response should be “Yes”.* | Yes | 1  *If Yes, go to A4* | A2 |
|  | No | 2 |  |
| Have you stopped drinking due to health reasons, such as a negative impact on your health or on the advice of your doctor or other health worker?  *This question is for those participants that did not drink during the past 12 months, but that have drunk in their lifetime.* | Yes | 1 *If Yes, go to A16* | A3 |
|  | No | 2 *If No, go to A16* |  |
| During the past 12 months, **how frequently** have you had at least one **standard alcoholic drink**?  *(READ RESPONSES, USE SHOWCARD)*  *For those that have consumed alcohol in the past 12 months.*  *A “standard drink” is the amount of ethanol contained in standard glasses of beer, wine, fortified wine such as sherry, and spirits. Depending on the country, these amounts will vary between 8 and 13 grams of ethanol. See showcard. For those participants that only consumed a few sips of alcohol during the past 12 months, the answer should be “Never”.* | Daily | 1 | A4 |
|  | 5-6 days per week | 2 |  |
|  | 3-4 days per week | 3 |  |
|  | 1-2 days per week | 4 |  |
|  | 1-3 days per month | 5 |  |
|  | Less than once a month | 6 |  |
|  | Never | 7 |  |
| Have you consumed any alcohol within the **past 30 days**?  *Select the appropriate response. Even if the participant has only consumed a few sips of alcohol in the past 30 days, the response should be “Yes”.* | Yes | 1 | A5 |
|  | No | 2 *If No, go to A13* |  |
| During the past 30 days, on how many **occasions** did you have at least one standard alcoholic drink?  *Ask the participant to think of the past 30 days only. Record the number of occasions. Note that there can be more than one occasion in which alcohol is consumed in a given day. For those participants that only drank a few sips of alcohol during the past 30 days, the answer should be “Zero” occasions.* | Number  Don't know 77 | └─┴─┘ *If Zero, go to A13* | A6 |
| During the past 30 days, when you drank alcohol, how many **standard** **drinks on average** did you have during one drinking occasion?  *(USE SHOWCARD)*  *Help the participant to average out the total number of drinks by using the showcard that shows standard alcoholic drinks.* | Number  Don't know 77 | └─┴─┘ | A7 |
| During the past 30 days, what was the **largest number** of standard drinks you had on a single occasion, counting all types of alcoholic drinks together?  *Ask the participant to think of the past 30 days only. This question is about the largest number of drinks that the participant had on one single occasion.* | Largest number  Don't Know 77 | └─┴─┘ | A8 |
| During the past 30 days, how many times did you have  **six or more** standard drinks in a single drinking occasion?  *Ask the participant to think of the past 30 days only, and to report the number of occasions when he/she had six or more standard drinks.* | Number of times Don't Know 77 | └─┴─┘ | A9 |

| **CORE: Alcohol Consumption, continued** | | | |
| --- | --- | --- | --- |
| **Question** | **Response** | | **Code** |
| During each of the **past 7 days**, how many standard drinks did you have each day?  *(USE SHOWCARD)*  *Don't Know 77*  *Ask the participant to think of each of the past 7 days. Use the showcard that shows standard alcoholic drinks to help the participant report the number of standard drinks for each of the past 7 days.*  *Record for each day the number of standard drinks. If no drinks record 0.* | Monday | └─┴─┘ | A10a |
|  | Tuesday | └─┴─┘ | A10b |
|  | Wednesday | └─┴─┘ | A10c |
|  | Thursday | └─┴─┘ | A10d |
|  | Friday | └─┴─┘ | A10e |
|  | Saturday | └─┴─┘ | A10f |
|  | Sunday | └─┴─┘ | A10g |
| I have just asked you about your consumption of alcohol during the past 7 days. The questions were about alcohol in general, while the next questions refer to your consumption of homebrewed alcohol, alcohol brought over the border/from another country, any alcohol not intended for drinking or other untaxed alcohol. Please only think about these types of alcohol when answering the next questions. | | | |
| During the **past 7 days**, did you consume any **homebrewed** alcohol, any alcohol **brought over the border/from another country**, any alcohol **not intended for drinking** or other **untaxed** alcohol? *(USE SHOWCARD)*  *[AMEND ACCORDING TO LOCAL CONTEXT]*  *Ask the participant to only think of homebrewed alcohol, any alcohol brought over the border/from another country, any alcohol not intended for drinking or other untaxed alcohol.* | Yes | 1 | A11 |
|  | No | 2 *If No, go to A13* |  |
| On average, **how many standard drinks** of the following did you consume **during the past 7 days**?  *[INSERT COUNTRY-SPECIFIC EXAMPLES]*  *(USE SHOWCARD)*  *Don't Know 77*  *Ask the participant to think of the past 7 days.*  *Use the showcard that specifies what standard drinks are for each type of alcohol. Alcohol not intended for drinking should be treated like spirits.*  *Record for each type of alcohol the number of standard drinks. If no drinks record 0.* | Homebrewed spirits, e.g. moonshine | **└─┴─┘** | A12a |
|  | Homebrewed beer or wine, e.g. beer, palm or fruit wine | **└─┴─┘** | A12b |
|  | Alcohol brought over the border/from another country | **└─┴─┘** | A12c |
|  | Alcohol not intended for drinking, e.g. alcohol-based medicines, perfumes, after shaves | **└─┴─┘** | A12d |
|  | Other untaxed alcohol in the country | **└─┴─┘** | A12e |

| **EXPANDED: Alcohol Consumption** | | | | | | | | | | | | | | | | | | | |
| --- | --- | --- | --- | --- | --- | --- | --- | --- | --- | --- | --- | --- | --- | --- | --- | --- | --- | --- | --- |
| During the **past 12 months,** how often have you found that you were not able to stop drinking once you had started?  *Ask the participant to think of the past 12 months. Read out all the answer options.* | | | | Daily or almost daily | | 1 | | | | | | | A13 | | | | | | |
|  |  |  |  | Weekly | | 2 | | | | | | |  |  |  |  |  |  |  |
|  |  |  |  | Monthly | | 3 | | | | | | |  |  |  |  |  |  |  |
|  |  |  |  | Less than monthly | | 4 | | | | | | |  |  |  |  |  |  |  |
|  |  |  |  | Never | | 5 | | | | | | |  |  |  |  |  |  |  |
| During the **past 12 months,** how often have you failed to do what was normally expected from you because of drinking?  *Ask the participant to think of the past 12 months. Read out all the answer options.* | | | | Daily or almost daily | | 1 | | | | | | | A14 | | | | | | |
|  |  |  |  | Weekly | | 2 | | | | | | |  |  |  |  |  |  |  |
|  |  |  |  | Monthly | | 3 | | | | | | |  |  |  |  |  |  |  |
|  |  |  |  | Less than monthly | | 4 | | | | | | |  |  |  |  |  |  |  |
|  |  |  |  | Never | | 5 | | | | | | |  |  |  |  |  |  |  |
| During the **past 12 months,** how often have you needed a first drink in the morning to get yourself going after a heavy drinking session?  *Ask the participant to think of the past 12 months. Read out all the answer options.* | | | | Daily or almost daily | | 1 | | | | | | | A15 | | | | | | |
|  |  |  |  | Weekly | | 2 | | | | | | |  |  |  |  |  |  |  |
|  |  |  |  | Monthly | | 3 | | | | | | |  |  |  |  |  |  |  |
|  |  |  |  | Less than monthly | | 4 | | | | | | |  |  |  |  |  |  |  |
|  |  |  |  | Never | | 5 | | | | | | |  |  |  |  |  |  |  |
| During the **past 12 months**, have you had family problems or problems with your partner due to **someone else’s** drinking?  *Ask the participant to think of the past 12 months. Read out all the answer options.*  *The participant should not think of his/her own drinking, but of someone else’s drinking.* | | | | Yes, more than monthly | | 1 | | | | | | | A16 | | | | | | |
|  |  |  |  | Yes, monthly | | 2 | | | | | | |  |  |  |  |  |  |  |
|  |  |  |  | Yes, several times but less than monthly | | 3 | | | | | | |  |  |  |  |  |  |  |
|  |  |  |  | Yes, once or twice | | 4 | | | | | | |  |  |  |  |  |  |  |
|  |  |  |  | No | | 5 | | | | | | |  |  |  |  |  |  |  |
| **CORE: Diet** | | | | | | | | | | | | | | | | | | |  |
| The next questions ask about the fruits and vegetables that you usually eat. I have a nutrition card here that shows you some examples of local fruits and vegetables. Each picture represents the size of a serving. As you answer these questions please think of a typical week in the last year. | | | | | | | | | | | | | | | | | | |  |
| **Question** | | | | **Response** | | | | | | | | | **Code** | | | | | |  |
| In a typical week, on how many days do you **eat fruit**?  *(USE SHOWCARD)*  *Ask the participant to think of any fruit on the showcard. A typical week means a "normal" week when the diet is not affected by cultural, religious, or other events. Ask the participant to not report an average over a period.* | | | | Number of days Don't Know 77 | | └─┴─┘ If Zero days, go to D3 | | | | | | | D1 | | | | | |  |
| How many **servings** of fruit do you eat on **one** of those days? (*USE SHOWCARD)*  *Ask the participant to think of one day he/she can recall easily. Refer to the showcard for serving sizes.* | | | | Number of servings  Don't Know 77 | | └─┴─┘ | | | | | | | D2 | | | | | |  |
| In a typical week, on how many days do you **eat vegetables**? *(USE SHOWCARD)*  *Ask the participant to think of any fruit on the showcard. A typical week means a "normal" week when the diet is not affected by cultural, religious, or other events. Ask the participant to not report an average over a period.* | | | | Number of days Don't Know 77 | | └─┴─┘ If Zero days, go to D5 | | | | | | | D3 | | | | | |  |
| How many **servings** of vegetables do you eat on one of those days? *(USE SHOWCARD)*  *Ask the participant to think of one day he/she can recall easily. Refer to the showcard for serving sizes.* | | | | Number of servings  Don’t know 77 | | └─┴─┘ | | | | | | | D4 | | | | | |  |
| **Dietary salt** | | | | | | | | | | | | | | | | | | |  |
| With the next questions, we would like to learn more about salt in your diet. Dietary salt includes ordinary table salt, unrefined salt such as sea salt, iodized salt, salty stock cubes and powders, and salty sauces such as soy sauce or fish sauce (see showcard). The following questions are on adding salt to the food right before you eat it, on how food is prepared in your home, on eating processed foods that are high in salt such as [insert country specific examples], and questions on controlling your salt intake. Please answer the questions even if you consider yourself to eat a diet low in salt.  *Read this opening statement out loud. Don't forget to use the showcard which will help the respondent when answering to the questions.* | | | | | | | | | | | | | | | | | | |  |
| How often do you **add salt or a salty sauce such as soy sauce** to your food right before you eat it or as you are eating it?  *(SELECT ONLY ONE)*  *(USE SHOWCARD)*  *Read out all the answer options. Use the showcard that shows salt and salty sauces.* | | | | | Always | | | | 1 | | | | | D5 | | | | |  |
|  |  |  |  |  | Often | | | | 2 | | | | |  |  |  |  |  |  |
|  |  |  |  |  | Sometimes | | | | 3 | | | | |  |  |  |  |  |  |
|  |  |  |  |  | Rarely | | | | 4 | | | | |  |  |  |  |  |  |
|  |  |  |  |  | Never | | | | 5 | | | | |  |  |  |  |  |  |
|  |  |  |  |  | Don't know | | | | 77 | | | | |  |  |  |  |  |  |
| How often is **salt, salty seasoning or a salty sauce added** in cooking or preparing foods in your household?  *Read out all the answer options. Select the appropriate response.* | | | | | Always | | | | 1 | | | | | D6 | | | | |  |
|  |  |  |  |  | Often | | | | 2 | | | | |  |  |  |  |  |  |
|  |  |  |  |  | Sometimes | | | | 3 | | | | |  |  |  |  |  |  |
|  |  |  |  |  | Rarely | | | | 4 | | | | |  |  |  |  |  |  |
|  |  |  |  |  | Never | | | | 5 | | | | |  |  |  |  |  |  |
|  |  |  |  |  | Don't know | | | | 77 | | | | |  |  |  |  |  |  |
| How often do you eat **processed food high in salt**? By processed food high in salt, I mean foods that have been altered from their natural state, such as packaged salty snacks, canned salty food including pickles and preserves, salty food prepared at a fast food restaurant, cheese, bacon and processed meat *[add country specific examples].*  *[INSERT EXAMPLES] (USE SHOWCARD)*  *Read out all the answer options. Use the showcard that shows processed food high in salt.* | | | | | Always | | | | 1 | | | | | D7 | | | | |  |
|  |  |  |  |  | Often | | | | 2 | | | | |  |  |  |  |  |  |
|  |  |  |  |  | Sometimes | | | | 3 | | | | |  |  |  |  |  |  |
|  |  |  |  |  | Rarely | | | | 4 | | | | |  |  |  |  |  |  |
|  |  |  |  |  | Never | | | | 5 | | | | |  |  |  |  |  |  |
|  |  |  |  |  | Don't know | | | | 77 | | | | |  |  |  |  |  |  |
| **How much salt or salty sauce** do you think you consume?  *Read out all the answer options and select the appropriate response.* | | | | | Far too much | | | | 1 | | | | | D8 | | | | |  |
|  |  |  |  |  | Too much | | | | 2 | | | | |  |  |  |  |  |  |
|  |  |  |  |  | Just the right amount | | | | 3 | | | | |  |  |  |  |  |  |
|  |  |  |  |  | Too little | | | | 4 | | | | |  |  |  |  |  |  |
|  |  |  |  |  | Far too little | | | | 5 | | | | |  |  |  |  |  |  |
|  |  |  |  |  | Don't know | | | | 77 | | | | |  |  |  |  |  |  |
| **EXPANDED: Diet** | | | | | | | | | | | | | | | | | |  | |
| **Question** | | | **Response** | | | | | | | | | **Code** | | | | |  | | |
| How important to you is **lowering the salt** in your diet?  *Select the appropriate response.* | Very important | | | | | | | 1 | | D9 | | | | |  | | | | |
|  | Somewhat important | | | | | | | 2 | |  |  |  |  |  |  | | | | |
|  | Not at all important | | | | | | | 3 | |  |  |  |  |  |  | | | | |
|  | Don't know | | | | | | | 77 | |  |  |  |  |  |  | | | | |
| Do you think that too much salt or salty sauce in your diet could cause a **health problem**?  *Select the appropriate response.* | Yes | | | | | | | 1 | | D10 | | | | |  | | | | |
|  | No | | | | | | | 2 | |  |  |  |  |  |  | | | | |
|  | Don't know | | | | | | | 77 | |  |  |  |  |  |  | | | | |
| Do you do any of the following on a regular basis to **control your salt intake**?  *(RECORD FOR EACH)*  *Select the appropriate response for each option. Ask the participant to only consider actions that he/she undertakes specifically to control salt intake, and not for any other purpose.* | | | | | | | | | | | | | | | |  | | | |
| Limit consumption of processed foods | | Yes | | | | | 1 | | | | D11a | | | | |  | | | |
|  |  | No | | | | | 2 | | | |  |  |  |  |  |  | | | |
| Look at the salt or sodium content on food labels | | Yes | | | | | 1 | | | | D11b | | | | |  | | | |
|  |  | No | | | | | 2 | | | |  |  |  |  |  |  | | | |
| Buy low salt/sodium alternatives | | Yes | | | | | 1 | | | | D11c | | | | |  | | | |
|  |  | No | | | | | 2 | | | |  |  |  |  |  |  | | | |
| Use spices other than salt when cooking | | Yes | | | | | 1 | | | | D11d | | | | |  | | | |
|  |  | No | | | | | 2 | | | |  |  |  |  |  |  | | | |
| Avoid eating foods prepared outside of a home | | Yes | | | | | 1 | | | | D11e | | | | |  | | | |
|  |  | No | | | | | 2 | | | |  |  |  |  |  |  | | | |
| Do other things specifically to control your salt intake | | Yes | | | | | 1  *If Yes, go to D11other* | | | | D11f | | | | |  | | | |
|  |  | No | | | | | 2 | | | |  |  |  |  |  |  | | | |
| Other (please specify) | | └─┴─┴─┴─┴─┴─┴─┘ | | | | | | | | | D11other | | | | |  | | | |

| **CORE: Physical Activity** | | | |
| --- | --- | --- | --- |
| Next I am going to ask you about the time you spend doing different types of physical activity in a typical week. Please answer these questions even if you do not consider yourself to be a physically active person.  Think first about the time you spend doing work. Think of work as the things that you have to do such as paid or unpaid work, study/training, household chores, harvesting food/crops, fishing or hunting for food, seeking employment. *[Insert other examples if needed].* In answering the following questions 'vigorous-intensity activities' are activities that require hard physical effort and cause large increases in breathing or heart rate, 'moderate-intensity activities' are activities that require moderate physical effort and cause small increases in breathing or heart rate.  *Read this opening statement out loud. It should not be omitted. The respondent will have to think first about the time he/she spends doing work (paid or unpaid work, household chores, harvesting food, fishing or hunting for food, seeking employment [Insert other examples if needed]), then about the time he/she travels from place to place, and finally about the time spent in vigorous as well as moderate physical activity during leisure time.*  *Remind the respondent when he/she answers the following questions that 'vigorous-intensity activities' are activities that require hard physical effort and cause large increases in breathing or heart rate, 'moderate-intensity activities' are activities that require moderate physical effort and cause small increases in breathing or heart rate. Don't forget to use the showcard which will help the respondent when answering to the questions.* | | | |
| **Question** | **Response** | | **Code** |
| **Work** | | | |
| Does your work involve vigorous-intensity activity that causes large increases in breathing or heart rate like *[carrying or lifting* *heavy loads, digging or construction work]*?  *[INSERT EXAMPLES] (USE SHOWCARD)*  *Ask the participant to think about vigorous-intensity activities at work only. Activities are regarded as vigorous intensity if they cause large increases in breathing and/or heart rate.* | Yes | 1 | P1 |
|  | No | 2  *If No, go to P 4* |  |
| In a typical week, on how many days do you do vigorous-intensity activities as part of your work?  *“Typical week” means a week when the participant is engaged in his/her usual activities. Valid responses range from 1-7.* | Number of days | └─┘ | P2 |
| How much time do you spend doing vigorous-intensity activities at work on a typical day?  *Ask the participant to think of a typical day he/she can recall easily in which he/she engaged in vigorous-intensity activities at work. The participant should only consider those activities undertaken continuously. Probe very high responses (over 4 hrs) to verify.* | Hours : minutes | └─┴─┘: └─┴─┘  hrs mins | P3 (a-b) |
| Does your work involve moderate-intensity activity, that causes small increases in breathing or heart rate such as brisk walking *[or carrying light loads]*?  *[INSERT EXAMPLES] (USE SHOWCARD)*  *Ask the participant to think about moderate-intensity activities at work only. Activities are regarded as moderate intensity if they cause small increases in breathing and/or heart rate.* | Yes | 1 | P4 |
|  | No | 2 *If No, go to P 7* |  |
| In a typical week, on how many days do you do moderate-intensity activities as part of your work?  *“Typical week” means a week when the participant is engaged in his/her usual activities. Valid responses range from 1-7.* | Number of days | └─┘ | P5 |
| How much time do you spend doing moderate-intensity activities at work on a typical day?  *Ask the participant to think of a typical day he/she can recall easily in which he/she engaged in moderate-intensity activities at work. The participant should only consider those activities undertaken continuously. Probe very high responses (over 4 hrs) to verify.* | Hours : minutes | └─┴─┘: └─┴─┘  hrs mins | P6 (a-b) |
| **Travel to and from places** | | | |
| The next questions exclude the physical activities at work that you have already mentioned.  Now I would like to ask you about the usual way you travel to and from places. For example to work, for shopping, to market, to place of worship. *[Insert other examples if needed]*  *The introductory statement to the following questions on transport-related physical activity is very important. It asks and helps the participant to now think about how they travel around getting from place-to-place. This statement should not* *be omitted.* | | | |
| Do you walk or use a bicycle *(pedal cycle)* to get to and from places?  *Select the appropriate response.* | Yes | 1 | P7 |
|  | No | 2  *If No, go to P 10* |  |
| In a typical week, on how many days do you walk or bicycle to get to and from places?  *“Typical week” means a week when the participant is engaged in his/her usual activities. Valid responses range from 1-7.* | Number of days | └─┘ | P8 |

| **CORE: Physical Activity, Continued** | | | |
| --- | --- | --- | --- |
| **Question** | **Response** | | **Code** |
| How much time do you spend walking or bicycling for travel on a typical day?  *Ask the participant to think of a typical day he/she can recall easily in which he/she engaged in transport-related activities. The participant should only consider those activities undertaken continuously. Probe very high responses (over 4 hrs) to verify.* | Hours : minutes | └─┴─┘: └─┴─┘  hrs mins | P9 (a-b) |
| **Recreational activities** | | | |
| The next questions exclude the work and transport activities that you have already mentioned.  Now I would like to ask you about sports, fitness and recreational activities (leisure) *[Insert relevant terms]*.  *This introductory statement directs the participant to think about recreational activities. This can also be called discretionary or leisure time. It includes sports and exercise but is not limited to participation in competitions. Activities reported should be done regularly and not just occasionally. It is important to focus on only recreational activities and not to include any activities already mentioned. This statement should not be omitted.* | | | |
| Do you do any vigorous-intensity sports, fitness or recreational *(leisure)* activities that cause large increases in breathing or heart rate like *[running or football]*?  *[INSERT EXAMPLES] (USE SHOWCARD)*  *Ask the participant to think about recreational vigorous-intensity activities only. Activities are regarded as vigorous intensity if they cause large increases in breathing and/or heart rate.* | Yes | 1 | P10 |
|  | No | 2  *If No, go to P 13* |  |
| In a typical week, on how many days do you do vigorous-intensity sports, fitness or recreational *(leisure)* activities?  *“Typical week” means a week when the participant is engaged in his/her usual activities. Valid responses range from 1-7.* | Number of days | └─┘ | P11 |
| How much time do you spend doing vigorous-intensity sports, fitness or recreational activities on a typical day?  *Ask the participant to think of a typical day he/she can recall easily in which he/she engaged in recreational vigorous-intensity activities. The participant should only consider those activities undertaken continuously. Probe very high responses (over 4 hrs) to verify.* | Hours : minutes | └─┴─┘: └─┴─┘  hrs mins | P12  (a-b) |
| Do you do any moderate-intensity sports, fitness or recreational *(leisure)* activities that cause a small increase in breathing or heart rate such as brisk walking*, [cycling, swimming, volleyball]*?  *[INSERT EXAMPLES] (USE SHOWCARD)*  *Ask the participant to think about recreational moderate-intensity activities only. Activities are regarded as moderate intensity if they cause small increases in breathing and/or heart rate.* | Yes | 1 | P13 |
|  | No | 2  *If No, go to P16* |  |
| In a typical week, on how many days do you do moderate-intensity sports, fitness or recreational *(leisure)* activities?  *“Typical week” means a week when the participant is engaged in his/her usual activities. Valid responses range from 1-7.* | Number of days | └─┘ | P14 |
| How much time do you spend doing moderate-intensity sports, fitness or recreational *(leisure)* activities on a typical day?  *Ask the participant to think of a typical day he/she can recall easily in which he/she engaged in recreational moderate-intensity activities. The participant should only consider those activities undertaken continuously. Probe very high responses (over 4 hrs) to verify.* | Hours : minutes | └─┴─┘: └─┴─┘  hrs mins | P15 (a-b) |

| **EXPANDED: Physical Activity** | | | |
| --- | --- | --- | --- |
| **Sedentary behaviour** | | | |
| The following question is about sitting or reclining at work, at home, getting to and from places, or with friends including time spent sitting at a desk, sitting with friends, traveling in car, bus, train, reading, playing cards or watching television, but do not include time spent sleeping.  *[INSERT EXAMPLES] (USE SHOWCARD)* | | | |
| How much time do you usually spend sitting or reclining on a typical day?  *Ask the participant to consider total time spent sitting at work, in an office, reading, watching television, using a computer, doing hand craft like knitting, resting etc. The participant should not include time spent sleeping.* | Hours : minutes | └─┴─┘: └─┴─┘  hrs mins | P16  (a-b) |

| **CORE: History of Raised Blood Pressure** | | | |
| --- | --- | --- | --- |
| **Question** | **Response** | | **Code** |
| Have you ever had your blood pressure measured by a doctor or other health worker?  *Ask the participant to only consider measurements done by a doctor or other health worker.* | Yes | 1 | H1 |
|  | No | 2 *If No, go to H6* |  |
| Have you ever been told by a doctor or other health worker that you have raised blood pressure or hypertension?  *Select the appropriate response.* | Yes | 1 | H2a |
|  | No | 2  *If No, go to H6* |  |
| Were you first told in the past 12 months?  *Only for those that have previously been diagnosed with raised blood pressure.* | Yes | 1 | H2b |
|  | No | 2 |  |
| In the past two weeks, have you taken any drugs (medication) for raised blood pressure prescribed by a doctor or other health worker?  *Ask the participant to only consider drugs for raised blood pressure prescribed by a doctor or other health worker.* | Yes | 1 | H3 |
|  | No | 2 |  |
| Have you ever seen a traditional healer for raised blood pressure or hypertension?  *Select the appropriate response.* | Yes | 1 | H4 |
|  | No | 2 |  |
| Are you currently taking any herbal or traditional remedy for your raised blood pressure?  *Select the appropriate response.* | Yes | 1 | H5 |
|  | No | 2 |  |

| **CORE: History of Diabetes** | | | |
| --- | --- | --- | --- |
| Have you ever had your blood sugar measured by a doctor or other health worker?  *Ask the participant to only consider measurements done by a doctor or other health worker.* | Yes | 1 | H6 |
|  | No | 2 *If No, go to H12* |  |
| Have you ever been told by a doctor or other health worker that you have raised blood sugar or diabetes?  *Select the appropriate response.* | Yes | 1 | H7a |
|  | No | 2  *If No, go to H12* |  |
| Were you first told in the past 12 months?  *Only for those that have previously been diagnosed with diabetes.* | Yes | 1 | H7b |
|  | No | 2 |  |
| In the past two weeks, have you taken any drugs (medication) for diabetes prescribed by a doctor or other health worker?  *Ask the participant to only consider drugs for diabetes prescribed by a doctor or other health worker.* | Yes | 1 | H8 |
|  | No | 2 |  |
| Are you currently taking insulin for diabetes prescribed by a doctor or other health worker?  *Ask the participant to only consider insulin that was prescribed by a doctor or other health worker.* | Yes | 1 | H9 |
|  | No | 2 |  |
| Have you ever seen a traditional healer for diabetes or raised blood sugar?  *Select the appropriate response.* | Yes | 1 | H10 |
|  | No | 2 |  |
| Are you currently taking any herbal or traditional remedy for your diabetes?  *Select the appropriate response.* | Yes | 1 | H11 |
|  | No | 2 |  |

| **CORE: History of Raised Total Cholesterol** | | | |
| --- | --- | --- | --- |
| **Questions** | **Response** | | **Code** |
| Have you ever had your cholesterol (fat levels in your blood) measured by a doctor or other health worker?  *Ask the participant to only consider measurements done by a doctor or other health worker.* | Yes | 1 | H12 |
|  | No | 2 *If No, go to H17* |  |
| Have you ever been told by a doctor or other health worker that you have raised cholesterol?  *Select the appropriate response.* | Yes | 1 | H13a |
|  | No | 2 *If No, go to H17* |  |
| Were you first told in the past 12 months?  *Only for those that have previously been diagnosed with raised total cholesterol.* | Yes | 1 | H13b |
|  | No | 2 |  |
| In the past two weeks, have you taken any oral treatment (medication) for raised total cholesterol prescribed by a doctor or other health worker?  *Ask the participant to only consider drugs for raised total cholesterol prescribed by a doctor or other health worker.* | Yes | 1 | H14 |
|  | No | 2 |  |
| Have you ever seen a traditional healer for raised cholesterol?  *Select the appropriate response.* | Yes | 1 | H15 |
|  | No | 2 |  |
| Are you currently taking any herbal or traditional remedy for your raised cholesterol?  *Select the appropriate response.* | Yes | 1 | H16 |
|  | No | 2 |  |

| **CORE: History of Cardiovascular Diseases** | | | |
| --- | --- | --- | --- |
| Have you ever had a heart attack or chest pain from heart disease (angina) or a stroke (cerebrovascular accident or incident)?  *Select the appropriate response.* | Yes | 1 | H17 |
|  | No | 2 |  |
| Are you currently taking aspirin regularly to prevent or treat heart disease?  *“Regularly” means on a daily or almost daily basis.* | Yes | 1 | H18 |
|  | No | 2 |  |
| Are you currently taking statins (Lovastatin/Simvastatin/Atorvastatin or any other statin) regularly to prevent or treat heart disease?  *“Regularly” means on a daily or almost daily basis.* | Yes | 1 | H19 |
|  | No | 2 |  |

| **CORE: Lifestyle Advice** | | | | | | |
| --- | --- | --- | --- | --- | --- | --- |
| **Question** | | **Response** | | | **Code** | |
| During the past 12 months, have you visited a doctor or other health worker? | Yes | | 1 | H20 | |  |
|  | No | | 2  *If No and C1=1, go to M1*  *If No and C1=2, go to CX1* |  |  |  |
| During any of your visits to a doctor or other health worker in the past 12 months, were you advised to do any of the following?  *(RECORD FOR EACH)*  *Select the appropriate response. Ask the participant to only consider advice from a doctor or other health worker.* | | | | | |  |
| Quit using tobacco or don’t start | Yes | | 1 | H20a | |  |
|  | No | | 2 |  |  |  |
| Reduce salt in your diet | Yes | | 1 | H20b | |  |
|  | No | | 2 |  |  |  |
| Eat at least five servings of fruit and/or vegetables each day | Yes | | 1 | H20c | |  |
|  | No | | 2 |  |  |  |
| Reduce fat in your diet | Yes | | 1 | H20d | |  |
|  | No | | 2 |  |  |  |
| Start or do more physical activity | Yes | | 1 | H20e | |  |
|  | No | | 2 |  |  |  |
| Maintain a healthy body weight or lose weight | Yes | | 1 | H20f | |  |
|  | No | | 2 |  |  |  |
| Reduce sugary beverages in your diet | Yes | | 1  *If C1=1 go to M1* | H20g | |  |
|  | No | | 2  *If C1=1 go to M1* |  |  |  |

| **CORE (for women only): Cervical Cancer Screening** | | | |
| --- | --- | --- | --- |
| The next question asks about cervical cancer prevention. Screening tests for cervical cancer prevention can be done in different ways, including Visual Inspection with Acetic Acid/vinegar (VIA), pap smear and Human Papillomavirus (HPV) test. VIA is an inspection of the surface of the uterine cervix after acetic acid (or vinegar) has been applied to it. For both pap smear and HPV test, a doctor or nurse uses a swab to wipe from inside your vagina, take a sample and send it to a laboratory. It is even possible that you were given the swab yourself and asked to swab the inside of your vagina. The laboratory checks for abnormal cell changes if a pap smear is done, and for the HP virus if an HPV test is done.  *Read this opening statement out loud. It should not be omitted.* | | | |
| Have you ever had a screening test for cervical cancer, using any of these methods described above?  *Select the appropriate response.* | Yes | 1 | CX1 |
|  | No | 2 |  |
|  | Don’t know | 77 |  |

| **Step 2 Physical Measurements** |
| --- |

| **CORE: Blood Pressure** | | | |
| --- | --- | --- | --- |
| Interviewer ID  *Record interviewer ID (in most cases interviewer would be the same as for behavioural measurements).* |  | └─┴─┴─┘ | M1 |
| Device ID for blood pressure  *Record device ID.* |  | └─┴─┘ | M2 |
| Cuff size used  *Select cuff size used.* | Small | 1 | M3 |
|  | Medium | 2 |  |
|  | Large | 3 |  |
| Reading 1  *Record first measurement after the participant has rested for 15 minutes. Wait 3 minutes before taking second measurement.* | Systolic ( mmHg) | └─┴─┴─┘ | M4a |
|  | Diastolic (mmHg) | └─┴─┴─┘ | M4b |
| Reading 2  *Record second measurement. Ask the participant to rest for another 3 minutes before taking the third measurement.* | Systolic ( mmHg) | └─┴─┴─┘ | M5a |
|  | Diastolic (mmHg) | └─┴─┴─┘ | M5b |
| Reading 3  *Record third measurement.* | Systolic ( mmHg) | └─┴─┴─┘ | M6a |
|  | Diastolic (mmHg) | └─┴─┴─┘ | M6b |
| During the past two weeks, have you been treated for raised blood pressure with drugs (medication) prescribed by a doctor or other health worker?  *Select appropriate response.* | Yes | 1 | M7 |
|  | No | 2 |  |
| **CORE: Height and Weight** | | | |
| **Question** | **Response** | | **Code** |
| **For women:** Are you pregnant?  *Pregnant women skip over height, weight, waist and hip measurements.* | Yes | 1 *If Yes, go to M16* | M8 |
|  | No | 2 |  |
| Interviewer ID  *Record interviewer ID (in most cases interviewer would be the same as for behavioural and blood pressure measurements).* |  | └─┴─┴─┘ | M9 |
| Device IDs for height and weight  *Record device IDs.* | Height | └─┴─┘ | M10a |
|  | Weight | └─┴─┘ | M10b |
| Height  *Record participant's height in cm with one decimal point.* | in Centimetres (cm) | └─┴─┴─┘. └─┘ | M11 |
| Weight  *If too large for scale 666.6*  *Record participant's weight in kg with one decimal point.* | in Kilograms (kg) | └─┴─┴─┘.└─┘ | M12 |
| **CORE: Waist** | | | |
| Device ID for waist  *Record device ID.* |  | └─┴─┘ | M13 |
| Waist circumference  *Record participant's waist circumference in centimetres with one decimal point.* | in Centimetres (cm) | └─┴─┴─┘.└─┘ | M14 |

| **EXPANDED: Hip Circumference and Heart Rate** | | | | | | |
| --- | --- | --- | --- | --- | --- | --- |
| Hip circumference  *Record participant's hip circumference in centimetres with one decimal point.* | | in Centimeters (cm) | | └─┴─┴─┘.└─┘ | | M15 |
| Heart Rate  *Record the three heart rate readings.* | | | | |  | |
| Reading 1 | Beats per minute | | └─┴─┴─┘ | | M16a | |
| Reading 2 | Beats per minute | | └─┴─┴─┘ | | M16b | |
| Reading 3 | Beats per minute | | └─┴─┴─┘ | | M16c | |
| **Step 3 Biochemical Measurements** | | | | | | |

| **CORE: Blood Glucose** | | | |
| --- | --- | --- | --- |
| **Question** | **Response** | | **Code** |
| During the past 12 hours have you had anything to eat or drink, other than water?  *It is essential that the participant has fasted.* | Yes | 1 | B1 |
|  | No | 2 |  |
| Technician ID  *Record ID of the person taking the measurement.* |  | └─┴─┴─┘ | B2 |
| Device ID  *Record device ID.* |  | └─┴─┘ | B3 |
| Time of day blood specimen taken (24 hour clock)  *Enter time measurement started.* | Hours : minutes | └─┴─┘: └─┴─┘  hrs mins | B4 |
| Fasting blood glucose  *[CHOOSE ACCORDINGLY: MMOL/L OR MG/DL]*  *Double check that the participant has fasted.* | mmol/l | └─┴─┘. └─┴─┘ | B5 |
|  | mg/dl | └─┴─┴─┘.└─┘ |  |
| Today, have you taken insulin or other drugs (medication) that have been prescribed by a doctor or other health worker for raised blood glucose?  *Select appropriate response.* | Yes | 1 | B6 |
|  | No | 2 |  |
| **CORE: Blood Lipids** | | | |
| Device ID  *Record device ID.* |  | └─┴─┘ | B7 |
| Total cholesterol  *[CHOOSE ACCORDINGLY: MMOL/L OR MG/DL]*  *Record value for total cholesterol.* | mmol/l | └─┴─┘. └─┴─┘ | B8 |
|  | mg/dl | └─┴─┴─┘.└─┘ |  |
| During the past two weeks, have you been treated for raised cholesterol with drugs (medication) prescribed by a doctor or other health worker?  *Select appropriate response.* | Yes | 1 | B9 |
|  | No | 2 |  |
| **CORE: Urinary sodium and creatinine** | | | |
| Had you been fasting prior to the urine collection?  *It is essential that the participant did not fast prior to urine collection.* | Yes | 1 | B10 |
|  | No | 2 |  |
| Technician ID  *Record technician ID.* |  | └─┴─┴─┘ | B11 |
| Device ID  *Record device ID.* |  | └─┴─┘ | B12 |
| Time of day urine sample taken (24 hour clock)  *Record time of day urine sample taken as reported by the participant.* | Hours : minutes | └─┴─┘: └─┴─┘  hrs mins | B13 |
| Urinary sodium  *Record value for urinary sodium.* | mmol/l | └─┴─┴─┘.└─┘ | B14 |
| Urinary creatinine  *Record value for urinary creatinine.* | mmol/l | └─┴─┘. └─┴─┘ | B15 |

| **EXPANDED: Triglycerides and HDL Cholesterol** | | | |
| --- | --- | --- | --- |
| Triglycerides  *[CHOOSE ACCORDINGLY: MMOL/L OR MG/DL]*  *Record value for triglycerides.* | mmol/l | └─┴─┘. └─┴─┘ | B16 |
|  | mg/dl | └─┴─┴─┘.└─┘ |  |
| HDL Cholesterol  *[CHOOSE ACCORDINGLY: MMOL/L OR MG/DL]*  *Record value for HDL cholesterol.* | mmol/l | └─┘. └─┴─┘ | B17 |
|  | mg/dl | └─┴─┴─┘.└─┘ |  |
