# Supplementary material for: Association between Angiotensin-converting enzyme (ACE) insertion/deletion polymorphism and hypertension in a Ghanaian population
Source: PLoS One. 2024 Dec 12;19(12):e0311692. doi: 10.1371/journal.pone.0311692 (PMC11637408; doi:10.1371/journal.pone.0311692)
Supplement: Attachment — Submitted filename: Response to Reviewers.doc [file pone.0311692.s005.doc]

Precious Bonney
University for Development Studies
Box TL 1350, Tamale

Dear Editor,

We wish to submit a revised version of the original research article entitled *“Association between Angiotensin-converting enzyme (ACE) insertion/deletion polymorphism and hypertension in a Ghanaian population]*” for consideration by PLOS ONE.

As per the corrections and suggestions requested on the aforementioned manuscript, the following changes have been made;

1. **Journal requirements:** All formatting requirements especially file naming errors have been corrected to meet PLOS ONE style requirements. Additionally, I can confirm that all relevant raw data including uncropped blot/gel results as well as the minimal data set have been uploaded as supplementary information files in this submission. The ethical considerations section have also been modified to include the IRB that approved the study and the information of the written consent. The supplementary figures were wrongly labeled, and the revised submission has been corrected to reflect the appropriate names of figures and supplementary files. A copy of the Fig-1 Gel and Fig 1 ACE referred to on page 7 and 12 respectively has been uploaded. The reference list has been curated to ensure currency and accuracy.
2. **Additional Editor comments:** The strobe cheklist per your request has been followed and uploaded as a supplementary file. The entire raw data has been made available as a supplementary file.
3. **Reviewer 1 Comments:** The limitations of the study have been included in the revised manuscript.
4. **Reviewer 2 Comments:** The abstract has been modified to increase the credibility of the article. However, the author appreciates the word limit according to PLOS ONE formatting style and therefore worked within those limits. Citations and references have also been checked throughout the document to rule out plagiarism. The statistical analysis section has been edited to include a more detailed description of the tools and statistical methods used. The p-values generated per the logistic regression model used to generate the results table 6 have been added to the table. The labels for figures and tables have been changed where necessary to provide more clarity on the corresponding tables and figures. The WHO step questionnaire has been uploaded as a supplementary file. The errors with regards to the number of categories of classification of weight categories used in the study is four and not five and has been modified to reflect same.The discussion section has been expanded to include more comparisons with similar studies in other populations.
5. Reviewer 2 minor corrections: Terminologies and acronyms which were previously not defined at first use have been corrected. Grammatical errors have been checked to improve readability. Page alignment and justification in the article have been corrected where necessary.

Please address all correspondence concerning this manuscript to me at [preciousbonney903@yahoo.com](mailto:preciousbonney903@yahoo.com)

Thank you for your consideration of this manuscript.

Precious Bonney
